# Titanium Degradation Products from Dental Implants: Mechanisms of Release, Biodistribution, and Analytical Detection in Biological Matrices

**DOI:** 10.3390/ijms27167346

**Published:** 2026-08-17

**Authors:** Łukasz Woźniak, Bożena Antonowicz, Żaneta Anna Mierzejewska, Ewelina Kosicka, Jérôme R. Lechien, Jan Borys

**Affiliations:** 1 Department of Dental Surgery, Medical University of Bialystok, M. Sklodowskiej-Curie 24A, 15-276 Bialystok, Poland; bozena.antonowicz@umb.edu.pl; 2 Institute of Biomedical Engineering, Faculty of Mechanical Engineering, Bialystok University of Technology, Wiejska 45C, 15-351 Bialystok, Poland; 3 Department of Information Technology and Robotics in Production, Faculty of Mechanical Engineering, Lublin University of Technology, Nadbystrzycka 36, 20-618 Lublin, Poland; e.kosicka@pollub.pl; 4 Department of Surgery, UMONS Research Institute for Health Sciences and Technology, University of Mons, Place du Parc 20, 7000 Mons, Belgium; jerome.lechien@umons.ac.be; 5 Department of Otolaryngology-Head & Neck Surgery, Foch Hospital, Paris Saclay University, 92150 Suresnes, France; 6 Department of Maxillofacial and Plastic Surgery, Medical University of Bialystok, M. Sklodowskiej-Curie 24A, 15-276 Bialystok, Poland

**Keywords:** titanium implants, tribocorrosion, titanium nanoparticles, biodistribution, peri-implant crevicular fluid, ICP-MS, biomaterial degradation, analytical methods

## Abstract

Titanium-based implants remain among the most successful biomaterials in modern dentistry, but accumulating evidence demonstrates that titanium is not biologically inert under long-term physiological conditions. Mechanical loading, tribocorrosion, acidic and oxidative microenvironments, and biofilm-driven electrochemical processes contribute to progressive surface degradation and the release of titanium ions, microparticles, and nanoparticles into surrounding tissues and biological fluids. The present narrative review synthesizes current evidence regarding the mechanisms of titanium release from dental implant surfaces, the biodistribution of degradation-derived species across local and systemic biological compartments, and the analytical methodologies used to detect and characterize them. Mechanisms of electrochemical corrosion, tribocorrosion, and nanoparticle generation are critically discussed alongside biofilm-mediated acceleration of surface degradation. The biological matrices in which titanium has been measured—including peri-implant tissues, peri-implant crevicular fluid, saliva, blood, regional lymph nodes, and distant organs—are reviewed with attention to the methodologies employed (ICP-MS, SP-ICP-MS, LA-ICP-MS, SEM-EDS, TEM, XRF, and synchrotron-based approaches) and their respective strengths and limitations. Methodological heterogeneity in implant characterization, sample preparation, contamination control, and discrimination between ionic and particulate species is identified as a major source of inconsistency across the current literature. The biological consequences of titanium dissemination—including oxidative, immunological, microbiome-associated, and systemic responses—are addressed in detail in a companion review. Together, these analyses are intended to support the design of more rigorous, integrated, and clinically translatable investigations of implant-associated biomaterial degradation.

## 1. Introduction

Titanium and titanium-based alloys have become foundational biomaterials in modern implantology and orthopedic reconstruction owing to their favorable strength-to-weight ratio, corrosion resistance, and ability to support osseointegration [1,2]. Since the pioneering work of Per-Ingvar Brånemark, titanium has traditionally been considered a biologically inert material capable of maintaining long-term stability within physiological environments. This assumption has shaped decades of clinical practice and biomaterial design. However, emerging evidence increasingly challenges the concept of complete titanium inertness, particularly under chronic functional loading and inflammatory conditions [3,4,5,6].

Implants are continuously exposed to a highly dynamic biological environment characterized by mechanical stress, electrochemical fluctuations, microbial colonization, salivary electrolytes, and immune-mediated oxidative processes. Under such conditions, titanium surfaces may undergo progressive degradation through corrosion, tribocorrosion, fretting, and wear-related mechanisms [7,8]. These processes lead to the release of titanium ions, oxide fragments, and micro- and nanoparticles into peri-implant tissues and systemic circulation [9,10]. The biological significance of titanium release depends not only on the chemical processes generating these species, but also on their subsequent biodistribution across biological compartments and on the analytical methodologies available to detect and characterize them [10,11,12].

Despite rapidly expanding literature, current evidence regarding titanium biodistribution remains fragmented across dentistry, orthopedics, toxicology, and materials science. Considerable heterogeneity exists with respect to analytical sensitivity, particle characterization, sampling protocols, and the ability to discriminate ionic titanium from particulate forms [13,14,15]. As a consequence, direct comparison across studies is frequently difficult, and conclusions regarding the magnitude and clinical significance of titanium release remain inconsistent.

The purpose of the present review is therefore to provide an integrated synthesis of the mechanisms through which titanium is released from dental implant surfaces, the biological matrices in which degradation-derived species have been detected, and the analytical methodologies underlying these measurements. Specific attention is given to the interplay between electrochemical corrosion, tribocorrosion, nanoparticle generation, and biofilm-mediated processes that drive surface degradation, as well as to methodological limitations that contribute to inconsistencies across the current literature.

### Search Approach

This article is a narrative review and did not follow a formal systematic-review protocol. Relevant literature was identified through searches of PubMed/MEDLINE, Scopus, and Web of Science covering the period from 2000 to May 2026, using combinations of the terms “titanium”, “titanium alloy”, “dental implant”, “corrosion”, “tribocorrosion”, “fretting”, “titanium particles”, “titanium nanoparticles”, “peri-implantitis”, “biodistribution”, “peri-implant crevicular fluid”, “saliva”, “blood”, “ICP-MS”, and related terms relevant to titanium release, biological distribution, and analytical detection. Reference lists of eligible primary studies and relevant reviews were additionally hand-searched to identify publications not retrieved through the electronic searches.

Although the principal database search was restricted to publications from 2000 onward, selected earlier studies were retained when they represented seminal or mechanistically important contributions to the field. In particular, older publications addressing fundamental aspects of titanium passivation, electrochemical corrosion, pH-dependent degradation, and fluoride-associated corrosion were included when they provided essential physicochemical foundations for interpreting more recent experimental and clinical evidence. Their inclusion was therefore based on their continuing mechanistic relevance rather than on publication date alone.

Studies were considered eligible when they investigated at least one of the following: mechanisms of titanium or titanium-alloy degradation; electrochemical corrosion, tribocorrosion, fretting, or mechanically induced particle release; local or systemic biodistribution of titanium-derived species; detection of titanium in biological matrices; biofilm-associated modification of titanium degradation; implant–abutment microgap- or loading-related processes relevant to degradation; or analytical techniques used for titanium quantification, localization, or particle characterization. Original in vitro, in vivo, and clinical investigations were prioritized. Systematic, scoping, and narrative reviews were used primarily to contextualize heterogeneous areas of evidence and to identify additional relevant primary studies. Orthopedic studies were included only when they provided directly transferable mechanistic or analytical information relevant to titanium degradation, particle transport, or systemic biodistribution. Non-English publications, conference abstracts without accessible full-text data, studies unrelated to titanium-based biomaterials, and publications without direct relevance to titanium release, biodistribution, or analytical detection were excluded.

The database searches identified 312 records, of which 241 remained after removal of duplicates. Titles and abstracts were screened for relevance, followed by full-text assessment of 117 publications. The final narrative synthesis included 90 publications. Reference lists of eligible primary studies and relevant reviews were additionally hand-searched to identify studies not retrieved through the initial electronic search.

Because the included evidence encompassed highly heterogeneous experimental, analytical, and clinical study designs, a single formal risk-of-bias instrument was not considered appropriate for all study types. Instead, the methodological robustness and interpretative value of individual studies were qualitatively appraised according to study design, sample size, presence and appropriateness of control groups, characterization of implant material and surface properties, definition of peri-implant health or disease, biological-matrix sampling procedures, contamination-control measures, analytical validation, reporting of limits of detection and quantification, ability to distinguish ionic from particulate titanium, and consideration of relevant clinical or environmental confounders.

When findings were conflicting, greater interpretative weight was assigned to studies with well-characterized patient populations or experimental conditions, validated analytical methodologies, rigorous contamination control, explicitly reported limits of detection and quantification, appropriate control groups, and clear characterization of the titanium material and biological matrix. Conflicting findings were retained and discussed rather than resolved according to the numerical predominance of studies, because methodological heterogeneity itself was considered a potentially important explanation for divergent results.

## 2. Mechanisms of Titanium Release and Implant Surface Degradation

Although titanium exhibits excellent corrosion resistance under physiological conditions, its long-term stability depends on the integrity of the passive titanium dioxide (TiO_2_) surface layer that spontaneously forms upon exposure to oxygen. This oxide film is thermodynamically stable and provides the basis for the material’s classical characterization as “biocompatible” and corrosion-resistant [16,17,18]. However, within the complex oral and peri-implant environment, the passive layer is continuously challenged by mechanical, electrochemical, inflammatory, and microbiological factors capable of destabilizing surface equilibrium and promoting degradation-related release of titanium-derived species [8,9,10,11,19,20,21,22].

A preliminary distinction is necessary, since “titanium” is not a single material. Dental implant fixtures are predominantly commercially pure titanium (cp-Ti, most often grade 4), whereas Ti-6Al-4V (grade 5) predominates in orthopedic components and appears in some abutments and prosthetic connections. The two behave differently in ways that matter here: the alloy releases aluminium and vanadium in addition to titanium, each carrying its own toxicological profile, and its multiphase α + β microstructure supports galvanic microcoupling that cp-Ti does not. Much of the mechanistic and biodistribution evidence synthesized below is drawn from both studies, and findings are not automatically transferable between them; where the distinction is material to interpretation, it is noted explicitly. The release of titanium degradation products is now understood as a multifactorial process involving simultaneous corrosion, tribocorrosion, fretting, wear, and biologically mediated surface alterations. Importantly, these processes rarely occur independently [5,6,7,8,9,10,19,23].

**Figure 1:**
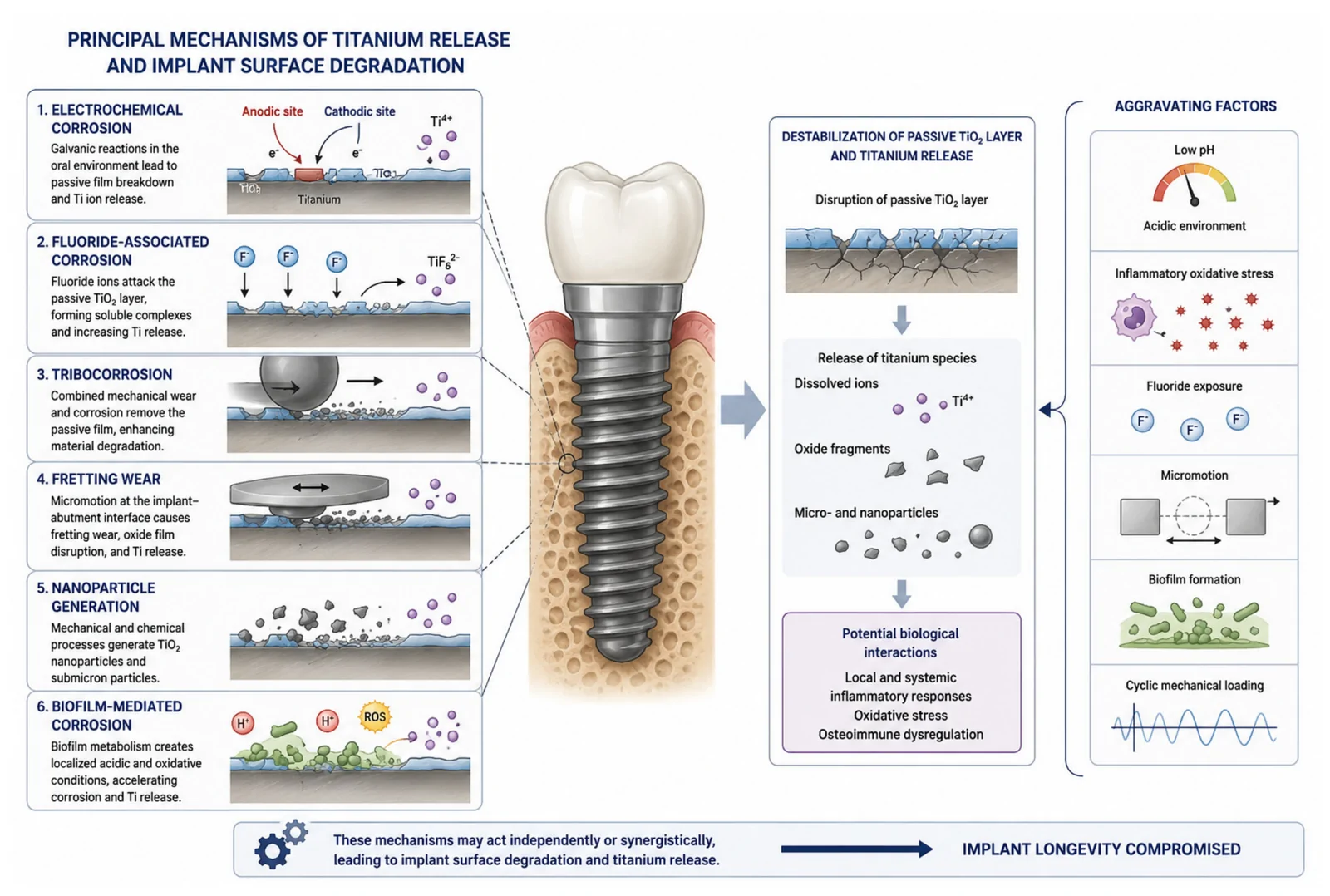
Mechanisms of titanium release and implant surface degradation. Schematic overview of the principal mechanisms contributing to titanium release from dental implant surfaces. Electrochemical corrosion, fluoride-associated corrosion, tribocorrosion, fretting wear, nanoparticle generation, and biofilm-mediated corrosion may act independently or synergistically to destabilize the passive TiO_2_ layer and promote the release of dissolved titanium ions, oxide fragments, and micro- and nanoparticles. Aggravating factors include low pH, inflammatory oxidative stress, fluoride exposure, micromotion, biofilm formation, and cyclic mechanical loading. Created in BioRender. Mierzejewska, A. (2026) https://BioRender.com/i5ve82v.

Instead, they interact dynamically, generating chemically and biologically heterogeneous degradation products ranging from dissolved ionic species to micro- and nanoparticles with distinct surface reactivity and immunological potential. The principal mechanisms of titanium release are summarized in Table 1 and schematically integrated in Figure 1, which illustrates how mechanical, electrochemical, inflammatory, fluoride-related, and biofilm-mediated factors converge on passive layer disruption and generation of titanium-derived degradation products [5,6,7,12,13,18].

### 2.1. Electrochemical Corrosion in Biological Environment

Titanium corrosion is fundamentally governed by disruption of the protective oxide layer and subsequent electrochemical reactions occurring at the implant surface. Although spontaneous repassivation generally occurs rapidly, repeated destabilization events may induce cumulative surface degradation over time. Corrosion susceptibility is strongly influenced by local environmental conditions, including acidic inflammatory microenvironments, fluoride-containing oral hygiene products, chloride-rich biological fluids, oxygen concentration gradients, bacterial metabolites, inflammatory oxidants, and galvanic interactions with restorative alloys [8,9,10,11,19,20,21,22,47].

Inflamed peri-implant tissues frequently exhibit reduced pH as a consequence of enhanced metabolic activity, neutrophil infiltration, and bacterial fermentation. Acidification destabilizes the TiO_2_ passive layer and accelerates ion dissolution kinetics, thereby increasing susceptibility to surface degradation. Fluoride ions may further exacerbate these processes through formation of soluble titanium–fluoride complexes that impair repassivation and promote progressive surface dissolution. Notably, the combined exposure to low pH and fluoride-rich environments appears particularly aggressive toward titanium surfaces and has repeatedly been associated with elevated corrosion currents in electrochemical investigations [19,20,21,22,29,30,31].

Reactive oxygen and nitrogen species generated during chronic inflammation also contribute substantially to corrosion-related processes. Activated neutrophils and macrophages release superoxide anions, hydrogen peroxide, hypochlorous acid, nitric oxide, and peroxynitrite, all of which may chemically modify oxide layers and promote localized surface breakdown. Consequently, peri-implant inflammation itself may establish a self-amplifying cycle in which inflammatory oxidative stress accelerates titanium degradation, while degradation-derived particles further enhance immune activation and ROS generation [24,25,26,27].

In addition, oral implants function within a highly conductive electrolyte-rich environment containing saliva, proteins, enzymes, and microbial metabolites capable of modifying electrochemical surface behavior. Protein adsorption may either stabilize or destabilize oxide films depending on protein composition and conformational interactions at the biomaterial interface. Albumin, mucins, and inflammatory proteins may further alter local electrochemical conditions and facilitate the formation of localized corrosion microdomains [24,25,26,27,28].

From an inorganic-chemistry perspective, titanium corrosion is governed by the dynamic equilibrium between anodic metal oxidation and formation of the protective oxide film. Metallic titanium may undergo anodic dissolution according to the simplified reaction Ti → Ti^4+^ + 4e^−^, whereas rapid oxidation and hydroxylation at the surface promote formation of a TiO_2_-dominated passive layer. Under favorable physiological conditions, this film substantially limits further metal dissolution. Its protective behavior, however, is strongly dependent on local electrochemical potential, oxygen availability, pH, and the presence of complexing species.

Acidification increases proton activity and facilitates defect formation and dissolution within the passive oxide. Fluoride becomes particularly relevant under acidic conditions because formation of hydrogen fluoride and soluble Ti(IV)-fluoride complexes shifts the surface equilibrium toward dissolution rather than repassivation. The process can be represented schematically by TiO_2_ + 6HF → H_2_TiF_6_ + 2H_2_O. Accordingly, the corrosive action of fluoride is strongly pH-dependent and should not be extrapolated from acidic, high-fluoride experimental conditions to routine exposure to neutral-pH oral-hygiene products. Local oxygen depletion, chloride accumulation, inflammatory oxidants, and crevice-like geometries may further impair repassivation and favor localized electrochemical attack. These effects are also material-dependent; commercially pure titanium and Ti-6Al-4V differ in microstructure, passive-film behavior, and the composition of released degradation products [19,20,21,22].

### 2.2. Tribocorrosion and Mechanically Induced Degradation

While corrosion alone contributes to titanium dissolution, clinical implant degradation is strongly influenced by tribocorrosion—the synergistic interaction between mechanical wear and electrochemical corrosion. Tribocorrosion is particularly relevant at implant–abutment interfaces, screw joints, and regions exposed to cyclic loading and micromotion, where repetitive mechanical stress continuously disrupts the protective oxide layer [7,8,10,11,12,32,33,34,35].

Mechanical destabilization of the passive film exposes underlying metallic titanium to direct electrochemical attack before complete repassivation can occur. Repeated loading cycles therefore induce continuous sequences of oxide destruction and reformation, ultimately resulting in progressive material loss and generation of degradation-derived particles. The severity of tribocorrosive degradation is influenced by multiple interconnected factors, including fretting wear associated with micromovements, cyclic fatigue loading, implant–abutment misfit, mastication-induced stress, microcrack formation, and surface fatigue occurring under inflammatory lubrication conditions [32,33,34,35,36].

The implant–abutment junction is considered one of the principal sources of titanium particle release in dental implant systems. Even minimal micromotions may generate substantial quantities of nanoscale debris over prolonged functional periods. Importantly, mechanically generated particles often exhibit irregular morphology, increased surface area, altered surface chemistry, and enhanced biological reactivity compared with bulk titanium surfaces, thereby potentially amplifying inflammatory and oxidative responses within peri-implant tissues [7,32,33,34,35,36].

Tribocorrosion may be further intensified under inflammatory conditions. Protein-rich exudates, altered lubrication properties, acidic pH, and bacterial biofilms significantly modify local friction coefficients and electrochemical behavior, thereby accelerating surface deterioration. Chronic inflammatory environments may therefore promote a self-perpetuating degradation cycle in which inflammation enhances tribocorrosion, while released particles further stimulate immune activation and oxidative stress. Within this framework, peri-implantitis may not represent solely a biofilm-driven infectious process, but rather a coupled biomechanical–electrochemical–immunological disorder involving continuous interactions between mechanical instability, biomaterial degradation, microbial dysbiosis, and host inflammatory responses [7,10,11,12,33,37].

#### Implant–Abutment Microgap, Micromotion, and Occlusal Loading

In two-piece implant systems, the implant–abutment junction should not be considered a mechanically static interface. Manufacturing tolerances, connection geometry, screw preload, prosthetic fit, and cyclic occlusal loading determine the dimensions and functional behavior of the microgap between connected components. Even when the nominal gap is small, repetitive mastication may produce reversible displacement, micromotion, and repeated opening and closing of the interface.

These movements are relevant to titanium degradation through two complementary mechanisms. First, repeated contact between the implant and abutment surfaces generates fretting, mechanically disrupts the passive TiO_2_ layer, and exposes underlying metal to renewed electrochemical attack before complete repassivation occurs. Second, cyclic changes in the microgap may generate a pumping effect that facilitates exchange of oral or peri-implant fluids across the implant–abutment interface. Once biological fluids and microbial products penetrate the connection, restricted oxygen exchange, accumulation of acidic metabolites, and altered ionic gradients may create a crevice-like electrochemical microenvironment that further favors localized corrosion [8,26,27].

Functional loading may therefore couple mechanical instability with microbial ingress and electrochemical degradation. Experimental studies demonstrating increased bacterial microleakage following dynamic loading support the concept that occlusal forces may influence not only mechanical complications but also the biological and electrochemical conditions within the implant–abutment junction [38]. The magnitude of this effect is expected to depend on connection design, machining precision, screw stability, loading direction, and the presence of parafunctional or excessive occlusal forces.

### 2.3. Titanium Nanoparticles and Biologically Reactive Degradation Products

One of the most important developments in contemporary implantology research is the recognition that titanium degradation generates substantial quantities of nano-scale particles with unique physicochemical and biological properties. Titanium nanoparticles may arise during multiple stages of implant placement and long-term function, including implant insertion, surface machining, tribocorrosion, polishing procedures, ultrasonic instrumentation, implantoplasty, and chronic mechanical wear associated with functional loading. As a result, peri-implant tissues are continuously exposed to complex populations of degradation-derived particulate debris that differ markedly from bulk titanium surfaces in both structure and biological behavior [5,6,7,12,13,15,38,39,40].

Particle size plays a critical role in determining biological activity. Nano-scale particles exhibit a markedly increased surface-area-to-volume ratio, enhanced catalytic reactivity, altered protein adsorption dynamics, greater cellular internalization capacity, and substantially higher oxidative potential compared with larger particles. These characteristics significantly increase their ability to interact with biological membranes, intracellular organelles, immune receptors, and redox-sensitive signaling pathways [12,13,38,39,40,41,42].

Following release, titanium nanoparticles may be internalized by macrophages, dendritic cells, fibroblasts, epithelial cells, and osteoblast-lineage cells through phagocytic and endocytic mechanisms. Intracellular accumulation has been associated with lysosomal destabilization, mitochondrial dysfunction, reactive oxygen species (ROS) amplification, inflammasome activation, and increased secretion of pro-inflammatory cytokines. Such responses suggest that nano-scale titanium debris may actively participate in chronic inflammatory signaling rather than functioning solely as passive byproducts of material degradation [12,13,38,41,42].

Importantly, titanium degradation products are chemically heterogeneous rather than compositionally uniform. Released species may include metallic titanium fragments, titanium dioxide particles, mixed oxide aggregates, alloy-associated elements, protein-coated particulate complexes, and ion-containing colloidal structures. Surface chemistry further influences biological activity, as oxidized particles may exhibit distinct catalytic and redox behavior depending on crystal structure, oxygen-vacancy distribution, surface charge, and adsorption of biological macromolecules. These factors likely contribute substantially to the variability observed across experimental studies investigating inflammatory, oxidative, and cytotoxic responses to titanium-derived particles [12,13,18,38,39,40,48,49].

Current evidence therefore supports the concept that titanium degradation products should not be regarded merely as inert wear debris, but rather as biologically active particulate mediators capable of modulating local tissue homeostasis, immune regulation, redox signaling, and potentially systemic inflammatory responses [12,13,38,39,40,41,42].

## 3. Biofilm-Mediated Acceleration of Titanium Release

Microbial colonization is traditionally regarded as a downstream phenomenon driven by implant surface exposure. However, the relationship between titanium surfaces and oral microorganisms is bidirectional: biofilms actively modify the electrochemical environment surrounding the implant and may substantially accelerate titanium release. From a release-mechanism perspective, biofilm-driven corrosion therefore represents an integral component of the same degradative process described in Section 2, rather than a separate phenomenon. The reciprocal biological consequences of titanium particles for microbial ecology are addressed in the companion review [8,9,10,11,12,23,37,43,44,45].

### Biofilm-Mediated Corrosion and Microbial Acceleration of Titanium Degradation

Microbial biofilms substantially modify the electrochemical environment surrounding titanium implants and play a critical role in regulating local corrosion-related processes. Mature biofilms create highly heterogeneous microenvironments characterized by localized pH reduction, oxygen depletion, metabolite accumulation, altered ionic gradients, enzymatic activity, and formation of extracellular polymeric matrices. These conditions may destabilize the protective titanium oxide layer and significantly accelerate corrosion-associated surface degradation [8,9,10,11,12,23,43,44,45,46].

Several periopathogenic microorganisms produce acidic metabolites and sulfur-containing compounds capable of directly influencing electrochemical behavior at titanium interfaces. As a consequence, inflammatory biofilms may promote passive layer destabilization, increased titanium ion release, enhanced tribocorrosion susceptibility, and generation of nano-scale degradation-derived particles. The biological effects of biofilms therefore extend beyond simple microbial colonization and involve direct modulation of biomaterial surface chemistry and electrochemical stability [43,44,45,46]

Bacterial endotoxins such as lipopolysaccharide (LPS) further contribute to this process by altering host immune responses and amplifying inflammatory oxidative stress. Increased ROS generation within chronically inflamed peri-implant tissues may indirectly accelerate corrosion processes through oxidative surface damage and impairment of repassivation mechanisms. Consequently, microbial dysbiosis and biomaterial degradation appear to interact through reciprocal amplification pathways rather than functioning as independent pathological events. It should be noted, however, that the effect of LPS on titanium electrochemistry is context-dependent rather than uniformly corrosive: experimental data indicate that LPS may accelerate corrosion at near-neutral pH yet attenuate it under the acidic conditions characteristic of active inflammation, such that the net influence of biofilm-derived endotoxin depends on the local pH and redox environment [23,41,42,43,44,45,46].

This establishes a potentially self-perpetuating cycle in which biofilm formation promotes local acidification and inflammation, thereby enhancing titanium degradation and particle release. Released particles subsequently amplify inflammatory signaling and oxidative stress, further destabilizing peri-implant ecological homeostasis and promoting progression of dysbiotic microbial communities [12,13,23,41,42,43,44,45,46].

Importantly, microbial effects on titanium surfaces are not restricted to classical electrochemical corrosion. Biofilm accumulation may also alter frictional and lubrication properties and modify local mechanical stress distribution at implant interfaces, thereby increasing susceptibility to tribocorrosive wear. In this respect, biofilm activity contributes to the same mechanically and electrochemically driven release described in Section 2, reinforcing the view of surface degradation as a process governed jointly by mechanical, electrochemical, and microbiological factors [10,11,12,23,33,37,43,44,45,46].

A further factor relevant to sustained release is that titanium-derived particles and bacterial products frequently act together rather than independently. Experimental work indicates that co-exposure to titanium particles and bacterial lipopolysaccharide (LPS) elicits a stronger inflammatory and oxidative response than either stimulus alone, so that even relatively low concentrations of titanium-derived particles may acquire biological significance when amplified by concurrent microbial activity. Because this amplification feeds back onto the electrochemical and tribocorrosive processes described above, microbial dysbiosis and biomaterial degradation can reinforce one another over time: dysbiotic communities accelerate surface breakdown, while the particles thereby released heighten the local response to bacterial products. The detailed intracellular signalling through which titanium particles and endotoxin converge is examined in the companion review; for the present purpose the key implication is mechanistic, namely that the generation of titanium-derived species should be understood as a continuous, biologically modulated process rather than a fixed material property.

The implant–abutment microgap provides a particularly relevant interface at which mechanical and microbiological factors may converge. Microorganisms and their metabolites can penetrate internal connection spaces and form protected microbial reservoirs that are less accessible to routine mechanical cleaning. Cyclic loading and micromotion may promote repeated fluid exchange across the junction, while loss of mechanical stability may enlarge or dynamically alter the effective microgap. This combination can increase bacterial microleakage and simultaneously promote fretting-related oxide disruption. Consequently, the microgap may function as both a microbial niche and a tribocorrosive interface, linking occlusal instability, biofilm persistence, localized inflammatory stimulation, and titanium degradation within a single mechanistic pathway.

## 4. Biodistribution of Titanium Degradation Products

The biological significance of titanium implant degradation depends not only on the generation of particles and ionic species, but also on their capacity to disseminate within local tissues and systemic compartments. Increasing evidence demonstrates that titanium-derived products are not confined to the implant surface. Instead, released ions, oxide fragments, and nano-/microparticles may accumulate in peri-implant tissues, enter vascular and lymphatic circulation, and distribute to distant organs. This biodistribution process represents a critical mechanistic bridge between local implant degradation and broader immunological or systemic effects [13,15,16,18,38,39,40,48,49,50].

Importantly, titanium dissemination is influenced by multiple variables, including particle size, morphology, surface chemistry, oxidation state, local inflammatory activity, implant design, duration of implantation, and mechanical loading conditions. Nano-scale particles appear particularly capable of translocation due to their enhanced mobility, cellular uptake, and ability to cross biological barriers [13,38,39,40,41,42,50].

**Figure 2:**
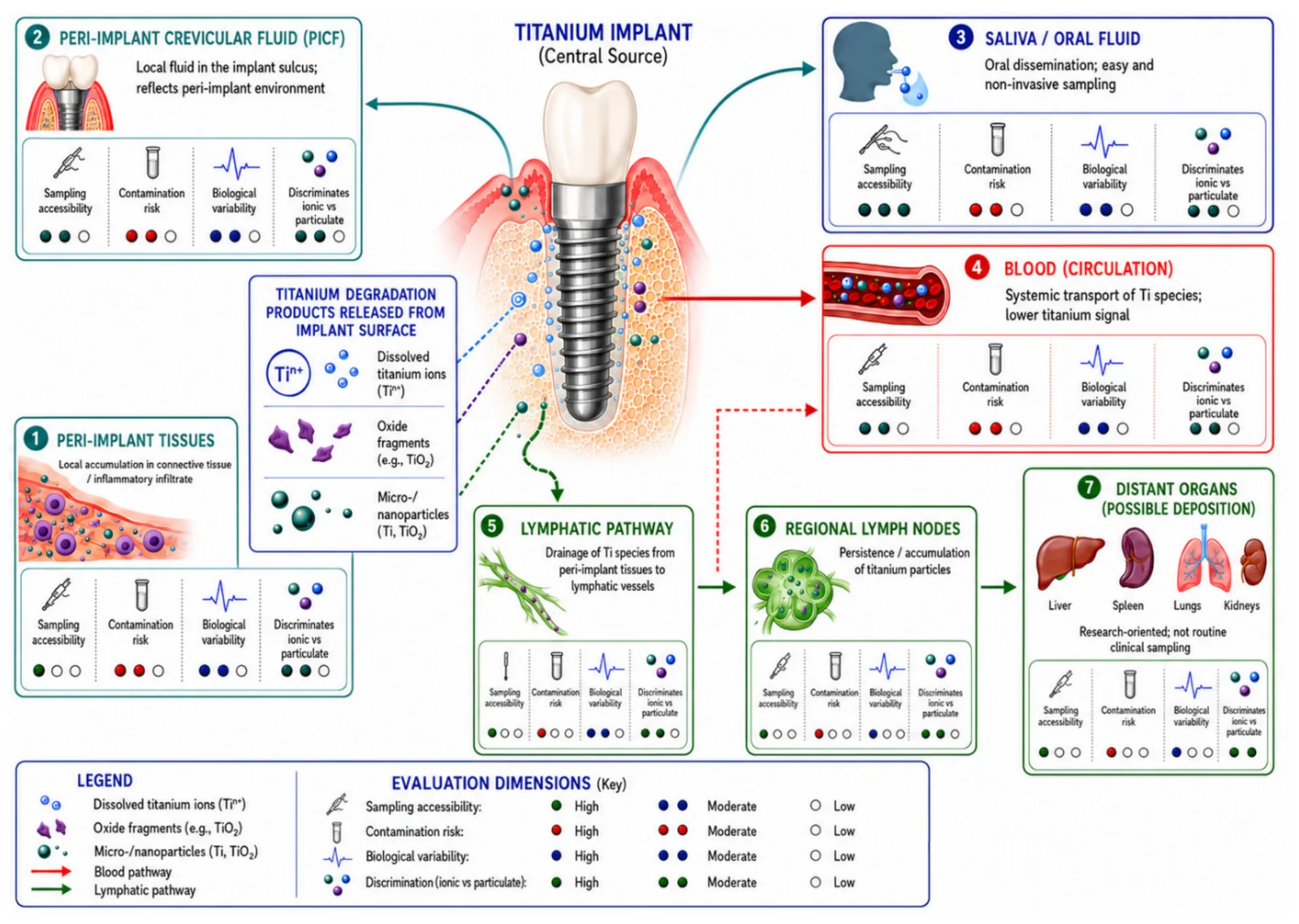
Biodistribution of titanium degradation products in biological matrices. Conceptual map of local and systemic dissemination pathways of titanium degradation products released from dental implant surfaces. Titanium ions, oxide fragments, and micro-/nanoparticles may accumulate in peri-implant tissues, enter peri-implant crevicular fluid and saliva, disseminate through blood and lymphatic pathways, and persist in regional lymph nodes and distant organs. The diagnostic and research value of each matrix is influenced by sampling accessibility, contamination risk, biological variability, and the ability to discriminate ionic from particulate titanium species. Created in BioRender. Mierzejewska, A. (2026) https://BioRender.com/dhj5266.

Despite growing literature, interpretation of titanium biodistribution remains complicated by substantial methodological heterogeneity. Different analytical techniques vary considerably in sensitivity, spatial resolution, contamination susceptibility, and ability to distinguish ionic titanium from particulate forms. Nevertheless, a consistent overall pattern has emerged indicating measurable titanium accumulation both locally and systemically following long-term implant exposure. The major biological compartments in which titanium degradation products have been detected are summarized in Table 2 and illustrated in Figure 2, which highlights the transition from local peri-implant deposition to biofluid transport, vascular dissemination, lymphatic translocation, and potential distant organ accumulation.

### 4.1. Local Accumulation Within Peri-Implant Tissues

Peri-implant tissues constitute the primary site of titanium particle deposition following implant surface degradation. Histological and ultrastructural investigations have repeatedly demonstrated the presence of titanium-containing debris within connective tissues adjacent to both dental and orthopedic implants, particularly under chronic inflammatory conditions. Titanium-derived particles have been identified within peri-implant mucosa, granulation tissue, peri-implant crevicular fluid, osteolytic lesions, bone marrow spaces, and multiple cellular populations, including macrophages, multinucleated giant cells, endothelial cells, and fibroblasts [13,15,16,18,54].

The concentration of titanium-containing particles is frequently elevated in tissues affected by peri-implantitis compared with clinically healthy peri-implant sites, suggesting a close association between inflammatory tissue destruction and accelerated biomaterial degradation. However, interpretation of this relationship remains complex, as inflammation itself may simultaneously intensify corrosion processes and particle release. Consequently, distinguishing whether titanium accumulation represents a primary pathogenic driver or a secondary consequence of peri-implant inflammation remains methodologically challenging [12,13,23,46,52,53,55,56,57].

Histopathological analyses commonly reveal dark particulate inclusions embedded within chronic inflammatory infiltrates rich in macrophages, lymphocytes, plasma cells, and foreign-body giant cells. Electron microscopy combined with energy-dispersive X-ray spectroscopy (SEM-EDS) has confirmed that many of these inclusions contain titanium and oxygen signatures consistent with titanium oxide-derived particulate debris. Such findings strongly support the biological persistence of degradation products within peri-implant tissues [16,18,52,53,55,56,57].

Particularly important is the frequent localization of titanium particles within macrophages, which indicates active phagocytic uptake and immune recognition of degradation-derived material. Intracellular accumulation may persist for prolonged periods and potentially sustain chronic inflammatory signaling through repeated activation of lysosomal stress pathways, mitochondrial dysfunction, and inflammasome-associated responses. This observation aligns closely with emerging osteoimmunological models proposing that titanium particles actively contribute to maintenance of inflammatory microenvironments rather than functioning solely as inert deposits [16,18,52,53,55,56,57].

Peri-implant crevicular fluid (PICF) has additionally emerged as a clinically relevant diagnostic matrix for evaluation of titanium release. Elevated titanium concentrations within PICF have been associated with peri-implant inflammation, increased probing depth, bleeding on probing, marginal bone loss, and implant surface deterioration. Consistent with this, dissolved titanium quantified by ICP-MS in submucosal plaque samples (a related but distinct peri-implant matrix) has been reported to be markedly higher at peri-implantitis sites than at healthy implants (0.85 ± 2.47 vs. 0.07 ± 0.19 after normalization for collected biomass; *p* = 0.033). Because PICF directly reflects biochemical conditions within the peri-implant microenvironment, it may represent a particularly valuable source for future biomarker-oriented investigations integrating titanium burden with oxidative stress mediators, inflammatory cytokines, and markers of osteoimmune dysregulation.

### 4.2. Salivary and Oral Dissemination Pathways

Saliva represents another clinically accessible biological compartment in which titanium release may be evaluated. Multiple studies have reported elevated salivary titanium concentrations in patients with dental implants, particularly in the presence of peri-implant inflammation, implant-supported full-arch rehabilitations, or prolonged functional loading. Salivary titanium may originate from direct corrosion products, tribocorrosive debris, biofilm-associated surface degradation, mechanical wear during mastication, and progressive deterioration at implant–abutment interfaces. Reported absolute concentrations, however, diverge to a degree that cannot be reconciled biologically, and the salivary literature is therefore best read as an illustration of the standardization problem rather than as a source of reference values. In a case–control study of 100 subjects, Papi et al. measured mean salivary titanium of 136.65 ± 263.28 µg/L in participants without implants, 489.60 ± 227.86 µg/L around clinically healthy implants, and 492.83 ± 313.90 µg/L at peri-implantitis sites, with a significant difference relative to controls (*p* < 0.001) but none between healthy and diseased implants (*p* > 0.05). A subsequent prospective cohort (n = 30) reported a severity-dependent gradient rising from 3.3 ± 1.49 µg/L (no implant) to 127 ± 13.32 µg/L (healthy implant) and 235.3 ± 17.94 µg/L (peri-implantitis; *p* = 0.001). The two datasets disagree both in absolute magnitude—control values differ roughly forty-fold between studies, in subjects who by definition carry no implant—and in their central conclusion regarding whether saliva discriminates healthy from diseased implants. Contamination control appears decisive in this respect: in a three-phase study incorporating helium collision-cell interference removal, Cionca et al. detected background titanium at approximately 0.18 µg/L in implant-free subjects, and after controlling dietary TiO_2_ intake all titanium and zirconium concentrations fell below the limit of quantification. Differences in collection method compound the problem, since passive drainage and absorbent-pad devices sample different salivary fractions and carry different contamination risks. Absolute salivary titanium values reported to date should therefore not be treated as benchmarks, and the direction of change across disease severity remains more defensible than any specific concentration [17,35,36,58,59,60,61].

The oral cavity constitutes a uniquely aggressive electrochemical environment characterized by fluctuating pH, enzymatic activity, microbial metabolites, salivary electrolytes, and continuous exposure to dietary compounds capable of influencing implant surface stability. Consequently, saliva may simultaneously function both as a medium facilitating corrosion-related processes and as a transport compartment for degradation-derived particles and ions released from implant surfaces [49,58,59,60,61,62].

Importantly, titanium-containing particles present within saliva may interact directly with oral microbial communities, epithelial barriers, and mucosal immune systems. Chronic low-level exposure to degradation-derived species may therefore contribute not only to local peri-implant inflammation, but also to broader alterations in oral ecological homeostasis. This concept is particularly relevant given emerging evidence suggesting reciprocal interactions between titanium particles, microbial dysbiosis, oxidative stress, and inflammatory signaling within peri-implant environments [19,49,58,59,60,61,62].

Despite its clinical accessibility and diagnostic potential, salivary titanium analysis remains methodologically challenging. Significant variability may arise from environmental contamination, dietary interference, differences in sampling protocols, inconsistent normalization strategies, and limited ability to distinguish dissolved ionic titanium from particulate forms. Moreover, salivary composition itself is highly dynamic and influenced by hydration status, circadian rhythm, oral hygiene practices, and inflammatory conditions, further complicating inter-study comparisons.

Standardization of salivary titanium assessment therefore remains an important unmet need within the field. Future studies integrating quantitative titanium analysis with inflammatory biomarkers, oxidative stress mediators, microbiome profiling, and detailed clinical phenotyping may substantially improve the translational value of saliva-based diagnostics in implantology research [40,42,49,64,65,66].

### 4.3. Systemic Dissemination and Distant Organ Accumulation

Perhaps the most controversial aspect of titanium biodistribution concerns the potential systemic dissemination of degradation-derived species. Increasing experimental and clinical evidence indicates that titanium particles and ions may enter circulatory and lymphatic pathways following release from implant surfaces, thereby extending biomaterial-associated interactions beyond the local peri-implant environment. Titanium has been detected in multiple systemic compartments, including whole blood, serum, plasma, urine, hair, regional lymph nodes, liver, spleen, lungs, kidneys, and bone marrow, supporting the concept that degradation-derived species are biologically mobile and capable of systemic distribution [40,42,49,64,65,66].

Direct evidence for distant-organ accumulation rests on a narrow experimental base. In a controlled rat model using double-focusing ICP-MS [40]. quantified titanium in organs and blood following implantation in the absence of wear, and studies tracking soluble and nanoparticulate titanium from implant debris by single-particle ICP-MS have since extended this to speciated measurement. Accumulation is reported particularly within reticuloendothelial tissues such as the liver and spleen. These organs are enriched in phagocytic macrophage populations responsible for sequestration and clearance of circulating particulate matter, suggesting that systemic titanium dissemination may preferentially involve immune-regulatory compartments associated with innate inflammatory surveillance.

Lymphatic dissemination appears especially relevant for nano-scale particles due to their enhanced mobility and cellular internalization capacity. Following phagocytosis or extracellular transport, titanium-containing particles may migrate toward regional lymph nodes, where persistent accumulation could theoretically contribute to chronic immune activation and low-grade inflammatory signaling. Histological studies demonstrating titanium-containing inclusions within lymphatic tissues further support the plausibility of long-term particle persistence beyond the immediate implant interface [40,42,49,64,65,66].

Nevertheless, blood-based titanium measurements in human studies remain highly inconsistent. While some investigations report significantly elevated circulating titanium levels in implant patients, others demonstrate only minimal or no detectable differences compared with control populations. This variability likely reflects substantial heterogeneity in implant number, cumulative surface area, duration of implantation, presence of peri-implant disease, analytical sensitivity, environmental background exposure, and contamination during sample preparation and analysis. Moreover, differences in sample processing and limited distinction between dissolved ionic species and particulate titanium further complicate interpretation of systemic measurements [18,48,49,58,59,60,61,62,64,65,66].

Importantly, detection of titanium within systemic compartments should not be interpreted as direct evidence of toxicity. Rather, these findings demonstrate that titanium-derived degradation products are capable of biological dissemination and interaction with systemic physiological systems. The clinical and biological significance of such dissemination remains incompletely understood and likely depends on cumulative particle burden, physicochemical characteristics of degradation products, host susceptibility, inflammatory status, and efficiency of systemic clearance mechanisms. Consequently, current evidence supports biological plausibility for systemic interactions without establishing definitive causal relationships between titanium dissemination and specific systemic pathological conditions.

### 4.4. Biological Implications of Titanium Biodistribution

The clinical significance of titanium dissemination likely extends beyond simple passive accumulation within tissues. Once distributed locally or systemically, degradation-derived species may interact dynamically with immune cells, extracellular matrices, endothelial barriers, and microbial ecosystems. The frequent localization of titanium-containing particles within macrophages and inflammatory infiltrates strongly suggests active biological participation in immune modulation rather than inert deposition alone [12,13,18,38,39,40,41,42,48,49,50].

Because the present review is concerned with release and biodistribution, these biological consequences are not examined in mechanistic detail here; they form the subject of the companion review. It is nonetheless useful to note that the compartments in which titanium has been detected—macrophage-rich peri-implant infiltrates, regional lymph nodes, and reticuloendothelial organs—are precisely those in which degradation-derived species would be expected to interact with immune and redox-regulatory systems. The pattern of biodistribution therefore itself suggests biological participation rather than purely passive deposition.

The cellular fate of internalised titanium debris—including the lysosomal, mitochondrial, and inflammasome-associated responses that may follow phagocytic uptake, and their links to osteoclastogenesis and connective tissue remodelling—is treated in the companion review and is therefore not expanded here.

As noted above, such causal relationships remain difficult to establish, and most current evidence demonstrates biological plausibility rather than direct pathological causation. Current evidence therefore supports a revised conceptual framework in which titanium implants should not be regarded as static inert devices, but rather as dynamic biomaterial systems continuously interacting with host immune, oxidative, and microbiological networks through the ongoing generation, transformation, and dissemination of biologically active degradation products.

## 5. Analytical Methods for Titanium Detection and Characterization

Interpretation of titanium biodistribution depends critically on the analytical methodologies used to detect and quantify degradation-derived species. The present section synthesizes the principal analytical approaches reported in the implantology and biomaterials literature, together with their major methodological limitations. These considerations are particularly relevant for the design of future biomarker-oriented investigations of titanium release [17,48,49,67].

### 5.1. Principal Analytical Approaches

Interpretation of titanium biodistribution critically depends on analytical methodology. One of the major limitations across the current literature is the lack of standardized detection protocols combined with insufficient physicochemical characterization of detected materials. Considerable variability exists not only in sample preparation and analytical sensitivity, but also in the ability of individual techniques to distinguish dissolved ionic titanium from particulate or aggregated forms. Consequently, direct comparison between studies remains challenging and may contribute substantially to inconsistencies reported in the literature [17,48,49,60,61,62,64,65,67].

Among currently available analytical approaches, inductively coupled plasma mass spectrometry (ICP-MS) is widely regarded as the most sensitive quantitative method for titanium measurement in biological samples. ICP-MS enables detection of trace titanium concentrations in blood, saliva, urine, and tissue digests with extremely high analytical sensitivity. However, despite its quantitative advantages, conventional ICP-MS generally cannot distinguish ionic titanium from particulate forms without additional fractionation or separation methodologies. A second constraint, less frequently acknowledged in the implantology literature, is spectral interference. Titanium has five stable isotopes, of which ^48^Ti is by far the most abundant (≈73.8%) and is therefore the preferred analyte; it is, however, isobarically overlapped by ^48^Ca, and additionally subject to polyatomic interference from species such as ^32^S^16^O and ^36^Ar^12^C. This matters acutely in the matrices most relevant to implantology, since saliva, peri-implant crevicular fluid, and mineralized tissue are all calcium-rich. The less abundant alternatives (^47^Ti, ≈7.4%; ^49^Ti, ≈5.4%) avoid the calcium overlap at a substantial cost in sensitivity. Contemporary practice addresses this through collision/reaction cell technology—typically helium in kinetic energy discrimination mode—or through high-resolution sector-field instruments, together with internal standardization to correct matrix effects and instrumental drift. Reported titanium concentrations obtained without such interference control, or without explicit description of the isotope monitored and the cell conditions employed, should be interpreted with caution, since uncorrected calcium overlap biases results upward in precisely those matrices where titanium is most often measured [16,17,48,49,67].

Laser ablation ICP-MS (LA-ICP-MS) has emerged as a particularly valuable technique for spatial mapping of titanium distribution within tissue sections. This approach enables visualization of elemental localization patterns within histological structures and may provide important insight into peri-implant accumulation and tissue-specific biodistribution. Similarly, scanning electron microscopy combined with energy-dispersive spectroscopy (SEM-EDS) is frequently employed for morphological visualization and elemental characterization of titanium-containing debris, particularly within peri-implant tissues, inflammatory infiltrates, and macrophage populations.

**Figure 3:**
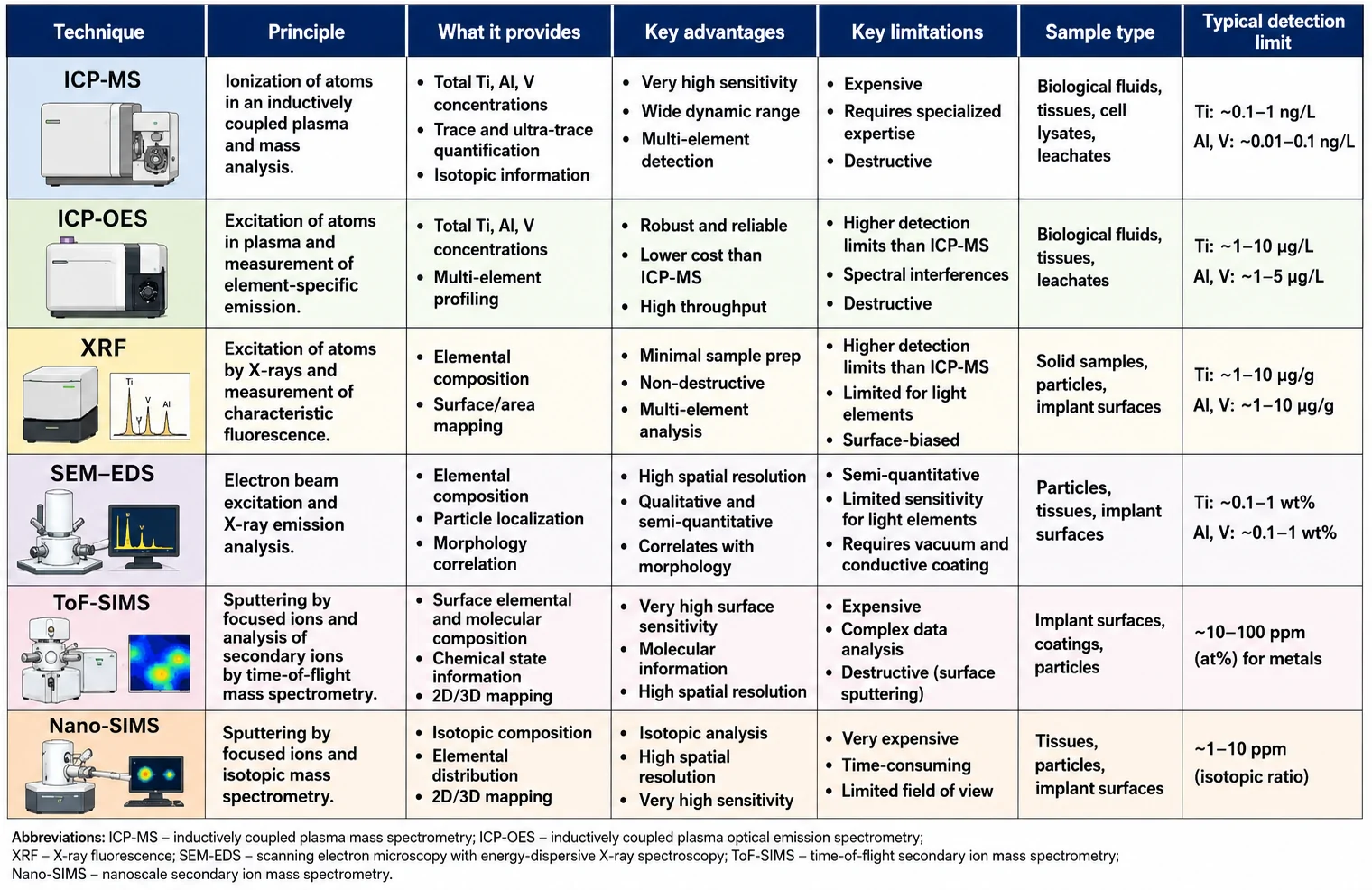
Analytical methods for titanium detection and characterization. Comparison of analytical techniques used for titanium detection and characterization in biological matrices. Conventional ICP-MS provides highly sensitive quantification of total titanium but does not distinguish ionic from particulate species without additional fractionation. SP-ICP-MS enables nanoparticle sizing and separation of soluble and particulate fractions, whereas LA-ICP-MS, SEM-EDS, TEM, XRF, and synchrotron-based methods provide complementary spatial, morphological, elemental, or chemical-state information. Integrated multimodal approaches are required to combine quantitative titanium burden with particle characterization, spatial localization, and contamination control. Created in BioRender. Mierzejewska, A. (2026) https://BioRender.com/dtzu81L.

Transmission electron microscopy (TEM) further enables ultrastructural visualization of nano-scale particles and intracellular localization, making it especially useful for investigating cellular internalization pathways and particle-associated organelle alterations. Additional methods such as X-ray fluorescence spectroscopy (XRF) allow elemental mapping within tissues with relatively limited sample preparation, whereas synchrotron-based imaging approaches provide exceptionally high-resolution elemental and chemical-state analyses capable of identifying complex particulate distributions within biological specimens. Approaches based on fractionation, most notably single-particle ICP-MS (SP-ICP-MS), have additionally been developed to discriminate the soluble ionic fraction from the nanoparticulate fraction within a single measurement, partially addressing the speciation limitations of conventional ICP-MS. Because no single analytical method fully captures titanium burden, particle morphology, spatial localization, and chemical state, the complementary strengths and limitations of currently available techniques are summarized in Table 3 and schematically compared in Figure 3 [17,48,49,60,61,62,64,65,67].

### 5.2. Inconsistencies and Limitations of Current Titanium Detection Methodologies

Analytical variability represents another major limitation within the current titanium-related literature and substantially complicates interpretation of biomaterial biodistribution and biological activity. Titanium quantification has been performed using a wide range of analytical techniques, including inductively coupled plasma mass spectrometry (ICP-MS), laser ablation ICP-MS (LA-ICP-MS), scanning electron microscopy with energy-dispersive spectroscopy (SEM-EDS), transmission electron microscopy (TEM), X-ray fluorescence spectroscopy (XRF), atomic absorption spectroscopy, and synchrotron-based imaging approaches. Although these technologies provide valuable insight into titanium localization and concentration, they differ considerably in analytical performance and biological interpretability.

Major methodological differences exist with respect to detection sensitivity, spatial resolution, susceptibility to contamination, ability to discriminate particle species, and sample preparation requirements. As a result, studies employing different analytical techniques may produce substantially divergent findings even when evaluating similar biological samples. Such variability significantly limits direct comparison between investigations and contributes to inconsistencies throughout the literature [17,48,49,60,61,62,64,65,67].

One of the most important analytical limitations is that many currently used methods quantify total titanium content without differentiating among dissolved ionic species, nanoparticles, microparticles, aggregated complexes, or biologically contaminated particulate debris. This distinction is biologically crucial because different titanium species may exhibit profoundly different physicochemical, immunological, and redox-associated behavior. Nano-scale particles, for example, possess substantially greater surface reactivity and cellular internalization capacity than larger aggregated structures, whereas dissolved ionic species may interact differently with proteins, extracellular matrices, and immune receptors.

Contamination further represents a major and often underappreciated challenge in trace-level titanium analysis. Titanium-containing compounds are ubiquitous in laboratory instruments, environmental dust, cosmetics, food additives, pharmaceuticals, and surgical equipment. Consequently, highly sensitive analytical systems may detect environmental or procedural contamination rather than biologically relevant implant-derived material. Despite this risk, contamination-control procedures are frequently insufficiently described or inconsistently standardized across studies. The magnitude of this effect is not hypothetical: in a controlled three-phase investigation, titanium and zirconium concentrations in oral mucosa fell below the limit of quantification once dietary TiO_2_ intake was restricted, indicating that exogenous ingestion rather than implant degradation may account for a substantial share of the signal attributed to biomaterial release. Reporting of limits of detection and quantification, procedural blanks, trace-metal-free consumables, and dietary control should therefore be regarded as minimum requirements rather than optional methodological detail [5,7,10,11,12,13,16,17,18,48,49,67].

These methodological limitations highlight the need for more rigorous analytical frameworks integrating advanced particle characterization, standardized sample preparation protocols, contamination-control procedures, and multimodal physicochemical analysis. Future studies capable of distinguishing specific titanium species and correlating them with inflammatory, oxidative, and clinical parameters will likely be essential for clarifying the true biological significance of titanium dissemination and biomaterial-associated inflammatory responses.

## 6. Methodological Limitations and Sources of Heterogeneity

Beyond the analytical limitations discussed in Section 5, several additional methodological factors contribute to substantial heterogeneity in the current literature on titanium release and biodistribution. These include variability in implant systems, the absence of standardized sampling protocols for biological matrices, and limited integration between microbiological and biomaterial analyses. These sources of heterogeneity, their consequences for cross-study comparability, and concrete parameters that future studies should report or harmonize are presented in Table 4.

### 6.1. Heterogeneity in Implant Systems and Surface Characteristics

One of the most significant challenges in interpreting titanium-related research is the extensive heterogeneity among implant systems and biomaterial surface characteristics. Current studies frequently combine implants that differ substantially in alloy composition, surface roughness, surface chemistry, oxide-layer thickness, manufacturing methods, connection geometry, loading conditions, and duration of implantation. Such variability introduces major confounding factors that complicate direct comparison between investigations and limit the ability to establish consistent mechanistic conclusions [5,6,7,8,9,11,12,32,33].

These implant-related variables strongly influence corrosion resistance, tribocorrosion susceptibility, nanoparticle generation, protein adsorption behavior, bacterial colonization patterns, and host immune responses. Even relatively subtle differences in surface topography or oxide composition may significantly alter electrochemical stability and biological interactions at the biomaterial–tissue interface. Consequently, the biological behavior of titanium implants should not be considered uniform across all implant systems [8,9,11,12,19,20,21,22].

For example, moderately roughened implant surfaces developed to enhance osseointegration provide increased surface area for bone attachment but may simultaneously create microtopographical niches more susceptible to corrosion processes, protein accumulation, and biofilm maturation. Likewise, implant–abutment connection geometry substantially influences micromotion, fretting wear, and tribocorrosive particle generation at mechanically stressed interfaces. Differences in prosthetic design and occlusal loading patterns may further modify long-term degradation dynamics and inflammatory responses [8,9,11,26,27,28,32,33].

Despite the importance of these parameters, many currently available clinical studies provide insufficient characterization of implant-specific variables. Inadequate reporting of alloy composition, surface treatment protocols, implant design, or loading conditions significantly limits reproducibility and weakens interpretation of biological findings. This methodological limitation may partially explain the considerable variability observed across studies investigating titanium release, peri-implant inflammation, and systemic dissemination [5,7,13,18,48,49].

Future investigations should therefore adopt more rigorous and standardized reporting frameworks incorporating detailed information regarding implant manufacturer and model, alloy composition, surface treatment methodology, implantation duration, prosthetic design, loading history, and maintenance protocols. Improved standardization of implant-related variables will likely be essential for establishing meaningful mechanistic relationships between biomaterial degradation, inflammatory signaling, and clinical outcomes in implantology research [5,7,13,16,17,48,49].

### 6.2. Lack of Standardized Biological Matrices and Sampling Protocols

Current studies evaluating titanium biodistribution investigate a wide range of biological compartments, including saliva, peri-implant crevicular fluid, blood, serum, plasma, urine, tissue biopsies, hair, and lymphatic tissues. Although such diversity reflects growing interest in both local and systemic titanium dissemination, it also introduces substantial methodological heterogeneity that complicates interpretation of findings and limits development of clinically meaningful reference standards [16,17,34,35,36,48,49,54].

At present, no consensus exists regarding optimal sampling protocols, normalization strategies, reference ranges, or clinically relevant titanium thresholds across biological matrices. Consequently, studies frequently employ highly variable collection and analytical methodologies, making direct comparison between datasets difficult and often preventing meaningful meta-analytic integration. This lack of standardization remains one of the major barriers to establishing reliable biomarker-based approaches for evaluation of implant-associated titanium exposure [16,17,35,36,48,49,54].

Salivary investigations are particularly heterogeneous due to variability in collection timing, stimulation methods, salivary flow rates, oral hygiene conditions, dietary contamination, and storage protocols. Because saliva composition is highly dynamic and influenced by multiple physiological and environmental factors, even relatively minor procedural differences may substantially alter measured titanium concentrations. In addition, difficulties distinguishing dissolved titanium species from particulate debris further complicate interpretation of salivary analyses [17,35,36,60,61].

Comparable methodological inconsistencies are observed in peri-implant tissue studies. Biopsy specimens frequently differ with respect to anatomical location, inflammatory severity, surgical collection procedures, tissue-processing protocols, and analytical preparation methods. Such variability may significantly influence detected titanium burden and inflammatory profiles, particularly given the highly heterogeneous distribution of particles within peri-implant lesions [13,15,16,18,48,49,54].

These methodological inconsistencies substantially limit inter-study comparability and reduce the reliability of broader conclusions regarding titanium biodistribution and biological significance. Future investigations will therefore require standardized sampling frameworks, harmonized analytical protocols, and well-defined normalization strategies capable of supporting reproducible cross-study comparisons and robust translational interpretation [16,17,48,49,66].

### 6.3. Limitations of Current Microbiome–Biomaterial Studies

Interpretation of peri-implant microbiome research remains substantially limited by methodological heterogeneity across currently available studies. Considerable variability exists in sampling strategies, analytical methodologies, implant systems, and clinical classification criteria, making direct comparison between investigations difficult and frequently preventing robust mechanistic conclusions. Many studies are additionally constrained by small cohort sizes, cross-sectional designs, inconsistent peri-implant disease definitions, inadequate control for confounding variables such as smoking and systemic inflammatory conditions, and limited characterization of implant surface properties or biomaterial status [23,43,44,45,46,71,72,73,74].

A further major limitation is the tendency to investigate microbial composition in isolation from the broader biological and physicochemical context of peri-implant tissues. Numerous studies characterize bacterial community structure without simultaneously evaluating titanium particle burden, oxidative stress biomarkers, electrochemical conditions, inflammatory mediators, or tribocorrosion status. As a consequence, mechanistic relationships linking microbiome alterations with biomaterial degradation and inflammatory signaling remain incompletely understood [23,43,44,45,46,71,72,73,74].

This reductionist approach may substantially underestimate the complexity of peri-implant disease, which likely emerges from dynamic interactions among microbial ecology, biomaterial degradation, immune activation, and redox dysregulation rather than from isolated microbiological shifts alone. Without integrated analysis of these interconnected pathways, it remains difficult to determine whether dysbiosis primarily drives peri-implant pathology, results from inflammatory tissue changes, or develops through reciprocal amplification with titanium-associated degradation processes [23,43,44,45,46,71,72,73,74].

Future research should therefore adopt more comprehensive systems-level approaches integrating metagenomics, metabolomics, redox profiling, quantitative titanium analysis, immune phenotyping, and detailed implant surface characterization within unified experimental and clinical frameworks. Longitudinal studies combining microbiome dynamics with biomaterial, inflammatory, and oxidative parameters may be particularly important for clarifying temporal relationships and identifying biologically meaningful pathways involved in peri-implant disease progression [23,43,44,45,46,71,72,73,74].

## 7. Future Directions for Biodistribution Research

Progress in the field will depend on moving beyond isolated descriptive observations toward integrated, biomarker-driven, and systems-level approaches capable of clarifying the temporal dynamics, physicochemical characteristics, and clinical significance of titanium release [17,48,49,71,72,73,74,75,76].

### 7.1. Biomarkers for Early Detection of Titanium-Associated Inflammatory Dysregulation

One of the major unmet clinical needs in implantology is the identification of sensitive biomarkers capable of detecting early peri-implant dysregulation before irreversible connective tissue destruction and bone loss occur. Current diagnostic approaches rely primarily on clinical probing depth, bleeding on probing, radiographic bone loss, and suppuration. However, these parameters generally reflect relatively advanced stages of disease progression rather than early biological imbalance [71,72,73,74,76,77].

Future biomarker strategies may therefore require integration of biomaterial-associated, inflammatory, oxidative, and osteometabolic indicators. Particularly promising targets include titanium concentrations within peri-implant crevicular fluid, oxidative stress biomarkers, inflammatory cytokines, lipid peroxidation products, mitochondrial dysfunction indicators, osteoclastogenic mediators, and microbiome-associated signatures. Among currently proposed candidates, IL-1β, TNF-α, the RANKL/OPG ratio, malondialdehyde (MDA), 4-hydroxynonenal (4-HNE), ROS-associated metabolites, oxylipins, and extracellular vesicle-associated biomarkers appear especially relevant [41,42,71,72,73,74,76,77].

Importantly, integration of redox-associated biomarkers with quantitative titanium analysis may provide substantially greater mechanistic insight into active peri-implant disease processes than conventional clinical measurements alone. Such multidimensional biomarker panels may ultimately facilitate earlier diagnosis, individualized risk assessment, and more precise monitoring of inflammatory progression [17,42,56,57,58,59,71,72,73,74,75,76].

### 7.2. Advanced Spatial and Elemental Imaging Technologies

Conventional bulk-tissue analyses provide only limited insight into the spatial and cellular complexity of peri-implant inflammation. Emerging high-resolution technologies now enable detailed characterization of particle localization, immune-cell heterogeneity, tissue-specific signaling pathways, and metabolic microenvironments within peri-implant tissues [48,49,77,78,79,80,81].

Particularly promising approaches include single-cell RNA sequencing, spatial transcriptomics, multiplex immunofluorescence, imaging mass cytometry, LA-ICP-MS tissue mapping, and nanoscale elemental imaging. These techniques may allow identification of macrophage subpopulations associated with titanium burden, localized oxidative stress niches, osteoclastogenic microdomains, and precise spatial relationships between degradation-derived particles and inflammatory lesions [67,68,69,70,75,78,79,80,81,82,83,84].

Such spatially resolved analyses may fundamentally improve mechanistic understanding of implant-associated tissue failure by revealing how inflammatory signaling networks, biomaterial degradation, and osteoimmune interactions are organized within peri-implant microenvironments [67,68,69,70,75,78,79,80,81,82,83,84].

### 7.3. Methodological Integration and Translational Priorities

To advance the field in a meaningful and clinically translatable manner, future research must prioritize substantially greater methodological integration, analytical rigor, and cross-disciplinary standardization. Current fragmentation between biomaterials science, microbiology, immunology, and clinical implantology limits mechanistic interpretation and hinders development of biologically coherent models of peri-implant disease [5,7,13,18,48,49,67,68,69,70,75,82,83,84].

One of the most urgent priorities is standardization of titanium analysis methodologies. Future investigations should adopt harmonized ICP-MS protocols, rigorous contamination-control procedures, detailed particle-size characterization, and advanced speciation analysis capable of distinguishing ionic titanium from nano-scale and aggregated particulate forms. Such standardization will be essential for improving reproducibility and enabling meaningful comparison across studies [48,49,67,68,69,70,75,82,83,84].

Equally important is the development of integrated clinical phenotyping frameworks incorporating standardized peri-implantitis definitions, reproducible radiographic criteria, comprehensive inflammatory biomarker panels, and detailed documentation of implant-system characteristics. Without consistent clinical characterization, interpretation of biomaterial-associated findings will remain limited by substantial cohort heterogeneity and confounding variability [71,72,73,74,76,77].

Future progress will also likely depend on implementation of mechanistic multi-omics approaches integrating microbiomics, lipidomics, metabolomics, transcriptomics, and redox-associated profiling. Such multidimensional analyses may provide critical insight into the complex interactions linking microbial dysbiosis, oxidative stress, immune regulation, and biomaterial degradation within peri-implant tissues. In particular, integration of redox biology and immunometabolic signaling may substantially improve understanding of host susceptibility and disease heterogeneity [71,72,73,74,76,77,78,79,80,81].

At the experimental level, more physiologically relevant systems are urgently needed. Advanced models incorporating dynamic tribocorrosion conditions, immune–biofilm co-cultures, clinically realistic nanoparticle exposure, and organoid or tissue-engineered platforms may better reproduce the complexity of peri-implant inflammatory microenvironments than currently available simplified in vitro systems [11,12,23,43,44,45,46,71,72,73].

**Figure 4:**
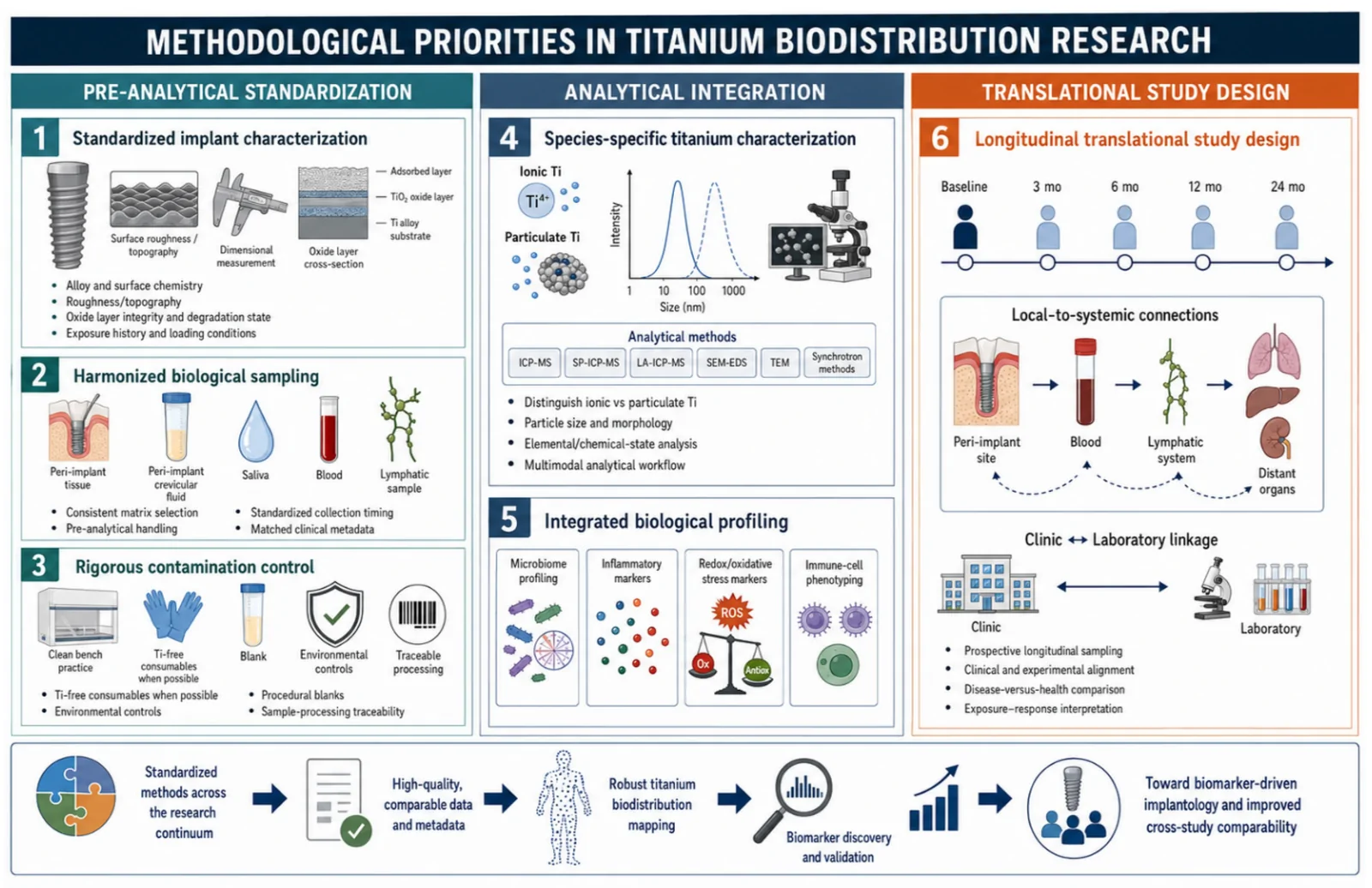
Future directions and methodological priorities in titanium biodistribution research. Recommended priorities for future studies investigating titanium release and biodistribution. Progress in the field requires standardized implant characterization, harmonized biological sampling, rigorous contamination control, species-specific particle characterization, integration of quantitative titanium analysis with microbiome, inflammatory, redox, and immune profiling, and longitudinal translational study designs. Addressing these methodological bottlenecks may support the development of biomarker-driven implantology and improve comparability across clinical and experimental studies. Created in BioRender. Mierzejewska, A. (2026) https://BioRender.com/1w69i0t.

Finally, longitudinal translational studies should become a central research priority. Prospective investigations integrating temporal titanium-release monitoring with inflammatory biomarker analysis, disease-progression tracking, and host-susceptibility profiling may be essential for clarifying causal relationships and identifying clinically meaningful predictive markers. Such approaches could ultimately facilitate transition from descriptive implantology toward a more mechanistically informed and personalized osteoimmunological framework for prevention, diagnosis, and management of peri-implant disease. A proposed roadmap for future titanium biodistribution research is presented in Figure 4. This framework emphasizes the need to move beyond isolated measurements of total titanium toward standardized, species-specific, longitudinal, and biologically integrated approaches [17,42,48,49,60,61].

## 8. Clinical Implications and Interpretation of Risk

The demonstration that titanium ions and particles can be released from dental implants should be interpreted in the context of the well-established long-term clinical performance of titanium implant systems. Long-term prospective observations, including 20-year follow-up data, demonstrate that titanium implants can remain functional for decades despite measurable material–tissue interactions and occasional biological or mechanical complications [85,86]. Therefore, evidence of titanium release does not invalidate the overall clinical biocompatibility or effectiveness of titanium-based implant therapy.

Equally important, analytical detection of titanium in saliva, peri-implant tissues, blood, lymphatic tissue, or distant organs demonstrates exposure and biological mobility but does not, by itself, demonstrate toxicity or clinically significant systemic disease. Human studies evaluating circulating titanium have reported small, variable, and in some cases statistically non-significant changes in systemic titanium levels [58,59,60,61,62,63,87]. The interpretation of trace-level measurements is additionally influenced by contamination, background environmental exposure, analytical sensitivity, and the limited ability of conventional total-element measurements to distinguish dissolved ionic titanium from particulate species [17,48,49,60,61,67].

The current evidence also does not establish a simple unidirectional causal pathway in which titanium dissemination independently causes peri-implantitis or systemic disease. Titanium-containing material can be detected in both healthy and diseased peri-implant tissues, whereas higher titanium burdens in peri-implantitis may reflect enhanced corrosion and wear occurring secondary to inflammation, biofilm activity, mechanical instability, or their combination. Thus, titanium degradation products may function as modifiers or amplifiers of an already dysregulated peri-implant environment rather than as an obligatory primary cause of disease.

This distinction has direct clinical relevance. At present, titanium concentration alone should not be interpreted as a diagnostic marker of peri-implantitis, systemic toxicity, or an indication for implant removal. No validated biological-matrix reference ranges or clinically meaningful toxicity thresholds have been established for implant-derived titanium. Conventional clinical assessment—including probing, bleeding on probing, suppuration, radiographic evaluation of marginal bone levels, assessment of prosthetic stability, and evaluation of local risk factors—therefore remains central to patient monitoring. Titanium analysis should currently be regarded primarily as a research tool or as part of selected mechanistic investigations rather than a stand-alone clinical diagnostic test.

From a preventive perspective, the mechanistic evidence reviewed here supports attention to factors capable of simultaneously increasing mechanical and electrochemical stress. Accurate implant–abutment fit, maintenance of screw stability, appropriate occlusal design, control of excessive or parafunctional loading, regular biofilm control, and management of peri-implant inflammation may reduce conditions promoting fretting, microgap-related leakage, and corrosion [32,33,34,35,36,85]. Similarly, the potential effects of fluoride should be interpreted according to concentration and local pH; available evidence does not justify generalized avoidance of routine fluoride-containing oral-hygiene products, but highly acidic and high-fluoride conditions may warrant particular consideration in susceptible clinical situations.

### Laser-Assisted Implant Surface Decontamination

Laser-assisted decontamination represents an additional clinical strategy relevant to the interaction between biofilm and titanium surfaces. Er and related laser systems have been investigated because they can reduce microbial contamination within complex implant surface topographies while limiting direct mechanical abrasion when appropriate irradiation parameters are used [88,89,90]. Their clinical effect, however, depends strongly on wavelength, energy density, pulse characteristics, irradiation time, cooling, and implant-surface morphology [83].

Randomized clinical investigations indicate that laser-assisted treatment may improve selected inflammatory outcomes, but current findings do not demonstrate universal superiority over conventional mechanical decontamination. In a randomized surgical study, both combined Nd–Er treatment and conventional mechanical instrumentation produced clinical improvement, whereas the laser protocol produced a greater reduction in bleeding on probing without a corresponding clear superiority in probing-depth reduction, attachment-level improvement, or RANKL/OPG modulation [89]. A separate randomized trial of Er:YAG irradiation likewise reported clinical and microbiological improvement without consistent superiority over mechanical debridement [88]. Laser treatment should therefore be considered an adjunctive decontamination modality rather than a replacement for established mechanical, surgical, and maintenance-based approaches.

The available evidence is even less sufficient to support routine prophylactic laser irradiation as a stand-alone method for preventing peri-implantitis. Its potential preventive value is more appropriately interpreted through improved biofilm management and surface decontamination in selected clinical situations. Standardization of laser parameters and long-term comparative trials are required before specific laser protocols can be recommended as universal preventive strategies.

## 9. Conclusions

Titanium-based implants remain highly biocompatible and clinically successful biomaterial systems, but they should not be regarded as completely chemically inert under all long-term physiological and pathological conditions [7,8,11,12,86]. Mechanical loading, tribocorrosion at implant–abutment interfaces, acidic and oxidative inflammatory microenvironments, biofilm-mediated electrochemical changes, and, under specific conditions, fluoride exposure may contribute to disruption of the passive surface layer and release of titanium-derived ions and particulate species [7,8,32,33]. Importantly, the occurrence of such release should be distinguished from demonstrated clinical toxicity: detection of titanium confirms biomaterial degradation and biological exposure but does not establish a causal relationship with peri-implant disease or systemic pathology [17,87]. Fluoride effects are conditional on low pH, and biofilm-derived endotoxin acts in a pH-dependent manner [19,20,21,22,23,43,44,45,46]. Once released, these degradation-derived species have been measured locally within peri-implant tissues and submucosal plaque [34,54], in saliva [35,36], and systemically in blood, lymphatic tissues, and distant organs [40,42,63]. The biological matrices and analytical methodologies through which such measurements are obtained vary considerably across studies. Conventional ICP-MS quantifies total titanium but does not resolve ionic from particulate species without fractionation, which single-particle ICP-MS provides [42,60,61]; spatially resolved mapping requires LA-ICP-MS [48,49]; and trace-level determination is vulnerable to spectral interference, notably ^48^Ca on ^48^Ti, unless collision-cell or high-resolution instrumentation is employed [17,67]. Together with inconsistent contamination control—exogenous TiO_2_ intake alone can account for much of the measured signal [17]—and heterogeneous sampling protocols, these factors continue to limit direct comparability across the current literature [16,66].

These observations support a revised view of titanium implants as dynamic biomaterial systems rather than static structural devices. Detection of titanium within biological compartments demonstrates dissemination and biological mobility but should not be interpreted as evidence of toxicity in itself. Establishing the biological and clinical significance of titanium release requires integrated approaches combining rigorous analytical chemistry, well-defined sampling protocols, and detailed clinical phenotyping [13,16,17,48,49,52,53,55].

The biological consequences of titanium dissemination—including oxidative, immunological, microbiome-associated, and systemic effects—are addressed in a companion review, which builds upon the mechanistic and analytical framework presented here. Together, these reviews are intended to support the design of more rigorous, integrated, and clinically translatable investigations of implant-associated biomaterial degradation, and to provide a foundation for the development of biomarker-oriented diagnostic strategies in implantology [13,16,17,48,49,71,72,73,74,75,76].

## Figures and Tables

**Table 1:** Mechanisms of titanium release and implant surface degradation.

Degradation Mechanism	Principal Driving Factors	Predominant Site	Dominant Product Type	Physicochemical/Biological Character	Aggravating Conditions	Refs
Electrochemical corrosion	Disruption of the passive TiO_2_ layer; acidic inflammatory microenvironment; chloride-rich fluids; oxygen gradients; galvanic coupling with restorative alloys; protein adsorption	Surfaces in contact with biological fluids; inflamed peri-implant regions; crevices	Dissolved Ti ions; soluble complexes; oxide breakdown products	Ionic species; interaction with proteins, ECM and immune receptors	Low pH; chloride; ROS/RNS; albumin/mucin adsorption altering oxide stability	[8,9,10,11,19,20,21,22,24,25,26,27,28]
Fluoride-associated corrosion	Formation of soluble Ti–fluoride complexes; HF formation at low pH; thinning/defecting of passive film	Surfaces exposed to fluoridated products; low-pH niches	Soluble Ti–F complexes; ions; nano-/microparticles	Impaired repassivation; large drops in polarization resistance reported, but under specific conditions—effects require the combination of low pH (≈≤3.5) with fluoride at concentrations typical of prophylactic gels (≳100 ppm F^−^), not those of everyday dentifrice use at neutral pH	Combined low pH + fluoride; prophylactic high-fluoride gels	[19,20,21,22,29,30,31]
Tribocorrosion	Synergy of mechanical wear and electrochemical corrosion; repeated oxide destruction–repassivation cycles	Implant–abutment interface; screw joints; cyclically loaded regions	Irregular micro- and nanoparticles with high surface area	Enhanced reactivity vs. bulk surface; altered surface chemistry; inflammatory potential	Fretting from micromotion; cyclic fatigue; implant–abutment misfit; mastication stress; inflammatory lubrication	[7,8,10,11,12,32,33,34,35,36,37]
Fretting/mechanical wear	Micromotion at mated components; surface fatigue; microcrack formation; component misfit	Implant–abutment junction; prosthetic connections	Metallic debris; oxide fragments. A systematic review reports 100 nm–54 µm across all release mechanisms and identification methods combined; the coarse end derives from implantoplasty and instrumentation, not from fretting at the connection, so procedure and mechanism should be specified whenever a size range is cited	Connection geometry strongly modulates ion release magnitude	Platform-matched vs. platform-switched design; occlusal overload; dissimilar abutment alloys	[32,33,34,35,36]
Nanoparticle generation (procedural & functional)	Implant insertion; surface machining; polishing; ultrasonic instrumentation; implantoplasty; chronic functional wear	Implant surface; surrounding tissues during clinical procedures	Nanoparticles; TiO_2_ particles; mixed oxide aggregates; protein-coated complexes	High surface-area-to-volume ratio; greater cellular uptake; higher oxidative potential	Surface chemistry; crystal structure; oxygen-vacancy distribution; instrumentation	[5,6,7,12,13,38,39,40,41,42]
Biofilm-mediated (microbial) corrosion	Acidic and sulfur-containing microbial metabolites; localized pH reduction; oxygen depletion; EPS matrix; enzymatic activity; LPS-driven inflammation	Biofilm-covered surfaces; peri-implant sulcus	Ti ions; nano-scale degradation debris	Context-dependent: LPS may accelerate (neutral pH) or inhibit (acidic pH) corrosion	Mature dysbiotic biofilm; endotoxin-amplified ROS; impaired repassivation	[23,43,44,45,46]

**Table 2:** Biological matrices in which titanium has been measured.

Biological Matrix	Clinical Accessibility	Diagnostic Value/Clinical Correlations	Reported Findings	Main Sources of Error/Contamination	Standardization Status	Refs
Peri-implant tissues (biopsy)	Invasive (surgical)	Direct evidence of local deposition; particles in macrophages, giant cells, endothelium, fibroblasts	Higher Ti burden in peri-implantitis vs. healthy sites; dark inclusions in inflammatory infiltrates. Tissue biopsies (ICP-MS): markedly higher Ti in peri-implantitis than control mucosa (98.7 ± 85.6 vs. 1.2 ± 0.9 µg/g). Submucosal plaque, reported separately (ICP-MS, DNA-normalized): 0.85 ± 2.47 vs. 0.07 ± 0.19 (*p* = 0.033); background in subjects without implants ≈ 0.18 µg/L (often < LOD; single samples 0.21–0.66 µg/L)	Heterogeneous particle distribution; variable site, severity, processing	No standardized protocol	[13,15,16,18,34,48,50,51,52,53,54]
Peri-implant crevicular fluid (PICF)	Non-invasive, site-specific	Reflects peri-implant microenvironment; candidate biomarker matrix	Elevated Ti associated with probing depth, BoP, and marginal bone loss. No dedicated quantitative PICF reference range yet established; matrix currently emerging/not standardized	Small volume; collection variability; ion vs. particle discrimination	Emerging; not standardized	[16,18,19,52,53,55,56,57]
Saliva	Non-invasive, readily accessible	Candidate severity marker, but discrimination between healthy and diseased implants is contested; transport compartment for debris	Findings conflict across studies. Papi et al. (ICP-MS, *n* = 100): 136.65 ± 263.28 µg/L (no implant) → 489.60 ± 227.86 (healthy) → 492.83 ± 313.90 (peri-implantitis); significant vs. control (*p* < 0.001) but healthy vs. diseased not significant (*p* > 0.05). Sharma et al. (ICP-MS, n = 30): 3.3 ± 1.49 → 127 ± 13.32 → 235.3 ± 17.94 µg/L; severity gradient significant (*p* = 0.001). Control values differ ≈ 40-fold between studies. Cionca et al. (He collision cell): background ≈ 0.18 µg/L; all values < LOQ after dietary TiO_2_ control	Dietary TiO_2_ (decisive—all values < LOQ once controlled); spectral interference (^48^Ca on ^48^Ti) unless collision cell used; collection method (passive drainage vs. absorbent pad); flow, circadian, hygiene variability	Not standardized; conflicting absolute values (≈40-fold) preclude reference ranges—major unmet need	[17,35,36,52,53,55,56,57,58,59,60,61]
Blood/serum/plasma	Minimally invasive	Indicator of systemic dissemination after long-term exposure	Circulating Ti reported to rise after placement and to be elevated in implant patients, but effects are typically small and frequently non-significant. Absolute values are not compared here because reporting units are inconsistent across studies (µg/L, ng/mL, and mass-normalized values are used interchangeably) and often incompletely specified	Trace-level contamination; environmental background; normalization	Inconsistent reference ranges	[49,58,59,60,61,62,63]
Regional lymph nodes	Invasive/post-mortem	Evidence of lymphatic translocation and regional accumulation	Ti deposition in submandibular/cervical/regional nodes (animal + human)	Limited accessibility; sampling/processing variability	Not standardized	[40,42,49,64,65,66]
Distant organs (liver, spleen, lung, kidney, brain)	Post-mortem/experimental	Demonstrates systemic biodistribution; long tissue half-lives	Ti in lung/liver/spleen/kidney months after placement; brain/spleen after implantoplasty	Confounded by environmental Ti; cross-sectional designs	Largely experimental	[40,42,49,64,65,66]

Notes: PICF = peri-implant crevicular fluid; BoP = bleeding on probing; MBL = marginal bone loss; LOD = limit of detection. Quantitative values are reported as mean ± SD as given in the cited primary studies. Units differ by matrix and are not directly comparable: biopsy tissue is expressed in µg/g ([54]), submucosal plaque is DNA-/biomass-normalized ([34]), and salivary concentrations are in µg/L ([36]). Serum/blood values are reported in inconsistent units across studies and changes are frequently non-significant, reflecting the methodological heterogeneity discussed in Section 5 and Section 6.

**Table 3:** Analytical methods for titanium detection and characterization.

Technique	Measured Parameter	Sensitivity/Resolution	Ion vs. Particle?	Speciation/Chemical State?	Main Limitations	Typical Matrix	Ref
ICP-MS	Quantitative total Ti (trace level)	Sub-µg/L in solution; no spatial resolution	No—needs fractionation	No	Cannot separate ionic from particulate Ti; ^48^Ca isobaric overlap on ^48^Ti requires collision/reaction cell or high resolution—critical in Ca-rich matrices (saliva, PICF, bone); contamination-prone; variable digestion	Blood, saliva, urine, tissue digests	[48,49,67,68,69,70]
SP-ICP-MS	Individual nanoparticle sizing + soluble fraction	High sensitivity; particle-by-particle counting	Yes—separates soluble vs. nanoparticulate	Partial (size, number)	Requires enzymatic/alkaline extraction; method optimization	Tissue extracts, fluids	[42,60,61]
LA-ICP-MS	Spatial elemental Ti mapping in sections	High sensitivity with localization	Partial—localization not speciation	No	Limited speciation; specialized instrumentation	Histological sections	[17,48,49]
SEM-EDS	Morphology + elemental composition of debris	Micro-scale; semi-quantitative	Partial—identifies particulate Ti + O signatures	No	Limited for trace ionic Ti; surface-sensitive; sampling bias	Peri-implant tissues, infiltrates, macrophages	[13,15,18,53]
TEM	Ultrastructural nanoparticle + intracellular imaging	Nano-scale resolution	Partial—visualizes particles	No	Very small sampling volume; labor-intensive; not quantitative for burden	Cells, ultrathin sections	[13,18,53]
XRF	Elemental mapping within tissues	Moderate resolution; limited prep	No—elemental signal only	No	Lower sensitivity than ICP-MS	Tissue specimens	[17,49,62,65]
Synchrotron (µ-SRXRF/XANES)	High-resolution elemental + chemical-state	Exceptionally high spatial; XANES resolves oxidation state, µ-SRXRF does not	XANES yes (chemical state); µ-SRXRF elemental only	Yes	Limited access; cost; complex handling	Whole organs, specimens	[62,64,65]
Atomic absorption spectroscopy	Quantitative total Ti	Lower sensitivity than ICP-MS; no spatial	No—total Ti only	No	Less sensitive at trace level	Digested samples	[67,68,69,70]

Notes: ICP-MS = inductively coupled plasma mass spectrometry; SP-ICP-MS = single-particle ICP-MS; LA = laser ablation; SEM-EDS = scanning electron microscopy with energy-dispersive spectroscopy; TEM = transmission electron microscopy; XRF = X-ray fluorescence; µ-SRXRF = micro synchrotron radiation XRF; XANES = X-ray absorption near-edge structure.

**Table 4:** Sources of methodological heterogeneity in titanium release and biodistribution research.

Category	Specific Problem	Consequence for Comparability	Concrete Parameters to Report/Harmonize	Refs
Implant systems & surface characteristics	Variability in alloy composition, surface roughness/chemistry, oxide thickness, manufacturing, connection geometry, loading, implantation duration; insufficient reporting	Major confounding; limits direct comparison and consistent mechanistic conclusions; weakens reproducibility	Manufacturer/model; alloy grade; surface treatment; oxide data; connection type; loading history; duration; maintenance	[5,6,7,8,9,11,12,26,27,28,32,33]
Sampling protocols for biological matrices	No consensus on collection, normalization, reference ranges, or clinically relevant thresholds; highly variable salivary and biopsy procedures	Difficult cross-dataset comparison; prevents meta-analytic integration; barrier to biomarker approaches	Standardized collection timing/stimulation; storage; normalization strategy; defined matrices and thresholds	[16,17,35,36,48,49,60,61]
Microbiome–biomaterial integration	Microbial composition studied in isolation from Ti burden, oxidative stress, electrochemical/tribocorrosion status; small, cross-sectional cohorts; inconsistent disease definitions	Mechanistic links between dysbiosis, degradation and inflammation remain unclear; underestimates disease complexity	Integrate metagenomics, metabolomics, redox profiling, quantitative Ti, immune phenotyping; longitudinal designs	[23,43,44,45,46,71,72,73,74]

## Data Availability

No new data were created or analyzed in this study. Data sharing is not applicable to this article.
